# Impact of the COVID-19 pandemic on household financial asset allocation: A China population study

**DOI:** 10.3389/fpsyg.2022.990610

**Published:** 2022-08-12

**Authors:** Hongwen Jia, Shugang Fan, Miao Xia

**Affiliations:** ^1^School of Economics, Lanzhou University, Lanzhou, China; ^2^School of Marxism, Lanzhou University, Lanzhou, China

**Keywords:** COVID-19, uncertainty, mental expectations, behavior selection, household financial asset allocation

## Abstract

During the COVID-19 pandemic, the Chinese government implemented a “dynamic zero” epidemic prevention policy, which led to an increase in the likelihood of business shutdowns, increased uncertainty about people's income, and changes in people's psychological expectations, which in turn influenced their behavioral choices. This study aims to understand the impact of COVID-19 and other major public health emergencies on household financial asset allocation. To do so, we conducted an online survey of 712 people in China to measure household financial asset allocation behavior during three different time periods: pre-pandemic, mid-pandemic, and post-pandemic. At the same time, we analyzed the impact of sociodemographic characteristics on risk attitudes and the differences in household asset allocation decisions at different pre-pandemic time points among people with different risk attitudes. The results show that household financial asset allocation changed significantly before, during, and after the pandemic, and residents' precautionary savings increased. In addition, gender, education level, occupation, and annual income have significant effects on risk preferences. The pandemic leads to increased uncertainty in economic and social development, people's psychological expectations of economic development play an important role in household financial asset allocation.

## Introduction

The COVID-19 epidemic has been sweeping the world for 3 years since late 2019, and several countries around the world have introduced public health measures to interrupt the spread of the epidemic during the pandemic, including social distancing (Aquino et al., 2020), border closures, quarantines, and mandatory masking (Tan et al., 2020). In China, the government has introduced a “dynamic zero” policy (Yang et al., 2022), which is a policy of restricting travel of people and temporarily suspending production of enterprises in a region when an outbreak occurs to stop the spread of the epidemic, resulting in a rapid zero of infected people at the social level. Although these public health measures have been effective in interrupting the spread of the epidemic, they have caused people in many countries around the world to experience restrictions on movement such as centralized or home quarantine, which may have some negative impact on the physical and mental health of the population (Savage et al., 2020; Cheikh Ismail et al., 2021; Faulkner et al., 2022).

The COVID-19 has had a huge impact on the macro economy (Barua, 2020), increasing the uncertainty of economic development (Baker et al., 2020) and negatively affecting international trade (Gruszczynski, 2020; Mena et al., 2022), financial markets (Sansa, 2020; Wang and Enilov, 2020; Zhang et al., 2020), industrial structures (Li et al., 2021) and labor markets (Forsythe et al., 2020; Rojas et al., 2020; Aum et al., 2021). Currently, academics do not define the timing of the post-epidemic era, but an important feature of the post-epidemic era is the recurrence of small-scale outbreaks (Li et al., 2022), which is consistent with the current situation of the epidemic in China; therefore, this paper defines 2022 and beyond as the post-epidemic era.

This paper focuses more on the impact of the COVID-19 epidemic on the micro-individual level. The COVID-19 epidemic exacerbates social development uncertainty and produces changes in individuals' psychological expectations of future economic and social development, leading to changes in their behavioral choices (Richard et al., 1996). Individuals are affected by negative news when faced with major contingencies such as COVID-19, causing investment sentiment and perceived uncertainty (Ali et al., 2020), while temporary business shutdowns and production stoppages cause shocks to the labor market, increasing individual labor income uncertainty (Khamis et al., 2021). Therefore, in the face of uncertainty about future scenarios, people's expectations about their income generate relatively pessimistic views, leading them to adopt more cautious and risk-averse choices when making investment decisions (Sha et al., 2022).

Expected utility theory assumes that markets are efficient and investors are perfectly rational, and that investors allocate assets based on expected utility maximization (Neumann and Morgenstern, 2007). The uncertainty of economic and social development breaks the perfect market assumption, and the influence of individual psychological expectations on household financial asset allocation behavior cannot be ignored (Gollier and Pratt, 1996; Brown et al., 2021). As uncertainty about future household income increases substantially, residents' precautionary saving incentives increase (Leland, 1968; Deaton, 1989), increasing the likelihood that households will change their portfolios (Yue et al., 2020).

Meanwhile, the impact of gender differences on risk attitudes is a stylized fact from economics and psychology studies: men are more willing to take risks than women and women are more cautious than men (Charness and Gneezy, 2012; Filippin and Crosetto, 2016). Also, differences in age (Bonsang and Dohmen, 2015), education (Rosen et al., 2003), occupation (Bonin et al., 2007), and annual income (Donkers et al., 2001) may produce different risk attitudes.

Most of the existing studies have focused on the macroeconomic impact of the COVID-19 epidemic, and most of the individual impact studies have focused on changes in consumption patterns (Pang et al., 2021), changes in travel patterns (Chinazzi et al., 2020), travel motivations (Sakai et al., 2021), travel motivations (Wachyuni and Kusumaningrum, 2020), and the impact of mobility restrictions on physical and mental health (Cullen et al., 2020; Savage et al., 2020), few studies have investigated household financial asset allocation, and this study helps explain the impact of the New Coronary Pneumonia epidemic on household financial asset allocation, enriching the related literature and provides a new perspective based on individual psychological expectations and risk attitudes to better understand the impact of the New Coronavirus epidemic on household financial asset allocation.

In this study we conducted an online survey to investigate how the Chinese population allocates household financial assets before (2019), during (2020–2021), and after (2022) the COVID-19 pandemic. Specifically, we surveyed 712 people from across China *via* the Internet and asked them about their household financial asset allocation at three different time periods using a questionnaire, while we assessed respondents' risk attitudes in order to determine their psychological expectations and analyzed the differences in household financial asset allocation behavior of people with different risk attitudes before, during, and after the pandemic and the differences in risk attitudes by different sociological demographic characteristics Impact.

## Materials and methods

### Study design and participants

A web-based cross-sectional survey study was conducted in China from June 25, 2022 to July 2, 2022. A total of 721 participants from across China were enrolled. The participant inclusion criteria were those living in China and aged ≥15 years. Participants were invited to take part in this research through the internet survey website: Questionnaire Star (Changsha Ranxing Information Technology Co., Ltd.) with a structured questionnaire using a random sampling method. These methods ensured the randomization of participants and also ensured that the questionnaire could be disseminated on a large scale.

The questionnaire was prepared in Chinese Word document format, and a pre-survey of 10 people was conducted before the large-scale release of the survey, with minor changes to the questions and wording to ensure the quality of the questionnaire data. A uniform resource locator (URL) was set up for the questionnaire, and the questionnaire was distributed both formally (website invitation to the respondent group) and informally (using social media platforms, such as WeChat, QQ, Knowledge Planet, etc.), with a 100% completion rate. Also, to ensure the accuracy of the respondents' answers, incentives were given to the respondents with monetary rewards for each questionnaire.

The questionnaire consists of four parts. The first part introduces the purpose of this survey, the second part asks respondents about their sociodemographic characteristics, including: gender, age, education level, marital status, occupation held, and annual income level, the third part assesses respondents' risk appetite and asks whether they believe that uncertainty in economic development will change their financial asset allocation behavior, and the fourth part consists of three questions (Table 1) designed to assess household financial asset allocation behavior during three different periods, i.e., pre-pandemic (2019), mid-pandemic (2020–2021), and post-pandemic (2022) for the COVID-19.

**Table 1:** Some questions in the questionnaire.

**Question**	**Answers**
How do you allocate your financial assets before, during and after the COVID-19? (Set total assets to 100 and allocate to the following financial products with no write-ins 0)	Current/fixed deposit
	Commercial insurance
	Stocks/funds
	Options/futures
	Bonds
	Overseas assets
	Precious metals

With the informed consent of the respondent, all data from the respondent will be used for this study only. In accordance with the privacy policy of Questionnaire Star, respondents' responses are confidential (https://www.wjx.cn/wjx/license.aspx?type=1) and respondents are not required to provide their names or contact details. In addition, respondents may stop participating in the survey at any point during the completion of the survey, if they do so their responses will not be saved, only if they click on the “submit” button, and if they complete the survey, they are considered to have voluntarily and uniformly participated in this anonymous study (Ammar et al., 2020).

### Data analysis and statistics

To ensure the validity of the data analysis, we used the Kolmogorov-Smirnov and Shapiro–Wilk tests for normality on the data (Lilliefors, 1967). Descriptive statistics were used to express the frequencies and percentages of responses for the definite class of variables. The effect of sociodemographic characteristics on risk preferences and risk attitudes was analyzed using chi-square tests in non-parametric tests. To assess whether there were significant differences in household financial asset allocation before and after the new crown pneumonia epidemic pandemic using Kruskal–Wallis test and multiple testing. Similarly, we used the Kruskal–Wallis test and multiple tests to analyze the differences in household financial asset allocation behavior of people with different risk attitudes before, during, and after the pandemic, and the above analyses were performed using SPSS version 28.0 (IBM, Chicago, IL, USA). Statistical significance was found at *p* < 0.05.

## Results

### Socio-demographic statistics

As shown in Table 2, male and female respondents accounted for 35.96 and 61.38%, respectively, with a higher proportion of female respondents than male; the age of respondents was concentrated between 20 and 39 years old, accounting for 89.47% of this range, close to 9/10 of the total sample, indicating that most of the respondents in this survey were young and middle-aged people, and therefore their education level was generally high. Four hundred and six of them (57.02%) had a bachelor's degree, 154 (21.63%) had graduated from a college or university, 67 (9.41%) had a master's degree or (9.55%) graduated from high school/junior college, and only 17 (2.39%) had a junior high school education or less. Of these, 374 (52.53%) were married, 272 (38.20%) were unmarried, 34 (4.78%) were divorced and 32 (4.49%) were widowed. The largest proportion of respondents' occupations was 295 (41.43%) for corporate employees, followed by 148 (20.79%) for students, 65 (9.13%) for institutions/civil servants, 63 (8.85%) for farmers, 62 (8.71%) for teachers, 45 (6.32%) for sole traders, 22 (3.09%) for retirees, and 12 (1.69%) for others, 1.69% were mostly flexibly employed. The distribution of annual income was as follows: 117 people (16.43%) had no income, 89 people (12.50%) had ≤ 10,000 yuan, 109 people (15.31%) had 10,001–30,000 yuan, 100 people (14.04%) had 30,001–50,000 yuan, 103 people (14.47%) had 50,001–80,000 yuan and 116 people (14.47%) had 80,001–120,000 yuan, 78 (10.96%) for ≥120,001 yuan.

**Table 2:** Sociodemographic characteristics of participants (*N* = 712).

**Items**	**Category**	**Frequency**	**Percentage (%)**
Gender	Male	275	38.62
	Female	437	61.38
Age (years)	15–19	37	5.20
	20–24	245	34.41
	25–29	202	28.37
	30–34	126	17.70
	35–39	64	8.99
	40–44	14	1.97
	45–49	9	1.26
	50–54	10	1.40
	55–59	2	0.28
	60–64	2	0.28
	≥65	1	0.14
Education level	Junior high school and below	17	2.39
	High school/Junior college	68	9.55
	College	154	21.63
	Undergraduate	406	57.02
	Master and above	67	9.41
Marital status	Unmarried	272	38.20
	Married	374	52.53
	Divorced	34	4.78
	Widowed	32	4.49
Occupation	Students	148	20.79
	Teachers	62	8.71
	Farmers	63	8.85
	Enterprise employees	295	41.43
	Individual merchants	45	6.32
	Public institution/civil servants	65	9.13
	Retirees	22	3.09
	Others	12	1.69
Annual income (yuan)	No income	117	16.43
	≤10,000	89	12.50
	10,001–30,000	109	15.31
	30,001–50,000	100	14.04
	50,001–80,000	103	14.47
	80,001–120,000	116	16.29
	≥120,001	78	10.96

### Respondents' attitudes toward risk

Investors' risk attitudes can be divided into three types: risk preferences, risk-neutral and risk-averse (Bodie et al., 2021), and we analyzed the risk attitudes of respondents through a questionnaire, of which 136 (19.1%) were risk preferences, 394 (55.3%) were risk-neutral, and 182 (25.6%) were risk-averse. At the same time, we asked respondents whether they would change their financial asset allocation behavior in the context of uncertain economic and social development (referred to as uncertainty change), and 536 of them (75.3%) gave positive answers, but 176 (24.7%) said they denied that they would change their financial asset allocation decisions because of economic and social uncertainty.

### Sociodemographic and risk attitudes

Table 3 shows whether different sociological demographic characteristics affect respondents' risk attitudes. The chi-square test demonstrates that differences in gender (*p* = 0.022), education (*p* = 0.001), occupation (*p* = 0), and annual income (*p* = 0.022) caused differences in risk attitudes, and age (*p* = 0.067) and marital status (*p* = 0.317) did not cause changes in risk attitudes.

**Table 3:** Sociodemographic and risk attitude variability analysis (*N* = 712).

**Items**	**Category**	**Risk attitude [*****n*** **(%)]**			***p*-value**
		**Risk preference**	**Risk-neutral**	**Risk-averse**	
Gender	Male	64 (47.06)	153 (38.83)	58 (31.87)	0.022^*^
	Female	72 (52.94)	241 (61.17)	124 (68.13)	
Age (years)	15–19	5 (3.68)	20 (5.08)	12 (6.59)	0.067
	20–24	50 (36.76)	132 (33.50)	63 (34.62)	
	25–29	41 (30.15)	125 (31.73)	36 (19.78)	
	30–34	23 (16.91)	69 (17.51)	34 (18.68)	
	35–39	10 (7.35)	27 (6.85)	27 (14.84)	
	40–44	2 (1.47)	7 (1.78)	5 (2.75)	
	45–49	3 (2.21)	4 (1.02)	2 (1.10)	
	50–54	1 (0.74)	8 (2.03)	1 (0.55)	
	55–59	0 (0.00)	0 (0.00)	2 (1.10)	
	60–64	1 (0.74)	1 (0.25)	0 (0.00)	
	≥65	0 (0.00)	1 (0.25)	0 (0.00)	
Education level	Junior high school and below	1 (0.74)	4 (1.02)	12 (6.59)	0.001^**^
	High school/Junior college	10 (7.35)	33 (8.38)	25 (13.74)	
	College	25 (18.38)	91 (23.10)	38 (20.88)	
	Undergraduate	86 (63.24)	229 (58.12)	91 (50.00)	
	Master and above	14 (10.29)	37 (9.39)	16 (8.79)	
Marital status	Unmarried	57 (41.91)	143 (36.29)	72 (39.56)	0.317
	Married	70 (51.47)	208 (52.79)	96 (52.75)	
	Divorced	5 (3.68)	25 (6.35)	4 (2.20)	
	Widowed	4 (2.94)	18 (4.57)	10 (5.49)	
Occupation	Students	25 (18.38)	75 (19.04)	48 (26.37)	0.000^**^
	Teachers	16 (11.76)	40 (10.15)	6 (3.30)	
	Farmers	12 (8.82)	40 (10.15)	11 (6.04)	
	Enterprise employees	62 (45.59)	176 (44.67)	57 (31.32)	
	Individual merchants	9 (6.62)	25 (6.35)	11 (6.04)	
	Public institution/civil servants	9 (6.62)	30 (7.61)	26 (14.29)	
	Retirees	1 (0.74)	6 (1.52)	15 (8.24)	
	Others	2 (1.47)	2 (0.51)	8 (4.40)	
Annual income (yuan)	No income	23 (16.91)	52 (13.20)	42 (23.08)	0.022^*^
	≤ 10,000	16 (11.76)	54 (13.71)	19 (10.44)	
	10,001–30,000	12 (8.82)	62 (15.74)	35 (19.23)	
	30,001–50,000	18 (13.24)	56 (14.21)	26 (14.29)	
	50,001–80,000	20 (14.71)	59 (14.97)	24 (13.19)	
	80,001–120,000	24 (17.65)	71 (18.02)	21 (11.54)	
	≥120,001	23 (16.91)	40 (10.15)	15 (8.24)	

* p < 0.05;

** p < 0.01.

### Sociodemographic and uncertainty change

Table 4 shows the difference between whether uncertainty in economic and social development changes household financial asset allocation decisions and sociodemographic characteristics. The chi-square test shows that different ages do not show significant differences with uncertainty change (*p* = 0.695), in addition, different genders (*p* = 0), education level (*p* = 0.001), marital status (*p* = 0), occupation (*p* = 0), and annual income (*p* = 0.004) show significant differences with uncertainty change.

**Table 4:** A chi-square test of whether sociodemographic and uncertainty change financial asset allocation decisions (*N* = 712).

**Items**	**Category**	**Uncertainty changes (** ***n*** **/%)**		**p-value**
		**Yes**	**No**	
Gender	Male	229 (42.72)	46 (26.14)	0.000^**^
	Female	307 (57.28)	130 (73.86)	
Age (years)	15–19	28 (5.22)	9 (5.11)	0.695
	20–24	184 (34.33)	61 (34.66)	
	25–29	156 (29.10)	46 (26.14)	
	30–34	91 (16.98)	35 (19.89)	
	35–39	52 (9.70)	12 (6.82)	
	40–44	10 (1.87)	4 (2.27)	
	45–49	6 (1.12)	3 (1.70)	
	50–54	7 (1.31)	3 (1.70)	
	55–59	1 (0.19)	1 (0.57)	
	60–64	1 (0.19)	1 (0.57)	
	≥65	0 (0.00)	1 (0.57)	
Education level	Junior high school and below	12 (2.24)	5 (2.84)	0.001^**^
	High school/Junior college	50 (9.33)	18 (10.23)	
	College	96 (17.91)	58 (32.95)	
	Undergraduate	324 (60.45)	82 (46.59)	
	Master and above	54 (10.07)	13 (7.39)	
Marital status	Unmarried	224 (41.79)	48 (27.27)	0.000^**^
	Married	291 (54.29)	83 (47.16)	
	Divorced	18 (3.36)	16 (9.09)	
	Widowed	3 (0.56)	29 (16.48)	
Occupation	Students	122 (22.76)	26 (14.77)	0.000^**^
	Teachers	44 (8.21)	18 (10.23)	
	Farmers	27 (5.04)	36 (20.45)	
	Enterprise employees	236 (44.03)	59 (33.52)	
	Individual merchants	36 (6.72)	9 (5.11)	
	Public institution/Civil servants	48 (8.96)	17 (9.66)	
	Retirees	16 (2.99)	6 (3.41)	
	Others	7 (1.31)	5 (2.84)	
Annual income (yuan)	No income	91 (16.98)	26 (14.77)	0.004^**^
	≤ 10,000	62 (11.57)	27 (15.34)	
	10,001–30,000	70 (13.06)	39 (22.16)	
	30,001–50,000	69 (12.87)	31 (17.61)	
	50,001–80,000	82 (15.30)	21 (11.93)	
	80,001–120,000	96 (17.91)	20 (11.36)	
	≥120,001	66 (12.31)	12 (6.82)	

*p < 0.05;

** p < 0.01.

### Household financial asset allocation before, during, and after the COVID-19

As expected, household savings increased during the COVID-19 (Table 5), with a median of 31 before the COVID-19, rising to 32 during the COVID-19, and the 75th percentile rising from 60 to 65.5, and then falling to 28.5 after the COVID-19.And then the Kruskal–Wallis test (Theodorsson-Norheim, 1986), only the stock/fund category (*p* = 0.029) was significantly different across time, while the other variables were not significantly different (Table 6). A multiple test for the stock/fund category computes the Bonferroni Adjusted *P*-value (Table 7), with significant differences before and during the COVID-19 (*p* = 0.032) and non-significant differences at other times.

**Table 5:** Median household financial asset allocation over time.

**Items**	**Median value (P25, P75)**		
	**Before**	**During**	**After**
	**COVID-19**	**COVID-19**	**COVID-19**
Current/fixed deposit	31 (16.25, 60)	32 (15, 65.5)	28.5 (14.60)
Commercial insurance	10 (0.20)	10 (0.20)	10 (0.20)
Stocks/funds	10 (0.20)	9 (0.18)	10 (0.18)
Options/futures	2 (0,12.75)	0 (0.12)	2 (0.12)
Bonds	3.5 (0.12)	3 (0.13)	3 (0.12)
Overseas assets	0 (0.9)	0 (0.10)	0 (0.10)
Precious metals	6 (0.19)	6 (0.20)	7 (0.20)

**Table 6:** Differences in household financial asset allocation by period.

**Items**	***K-W*-test statistic *H*-value**	***P*-value**
Current/fixed deposit	0.970	0.616
Commercial insurance	1.088	0.581
Stocks/funds	7.093	0.029
Options/futures	1.563	0.458
Bonds	0.232	0.89
Overseas assets	0.174	0.917
Precious metals	0.218	0.897

**Table 7:** Stock/fund class multiple testing.

**Items**	***P*-value**	**Bonferroni Adjusted *P*-value**
During-after COVID-19	0.522	1.000
Before-during COVID-19	0.011	0.032
Before-after COVID-19	0.055	0.165

### Risk attitude and household financial asset allocation

The Kruskal–Wallis test (*H*-value) study was used to analyze the differential impact of different risk attitudes on household financial asset allocation, and the results are shown in Table 8. Different risk attitudes produced statistically significant differences in all household asset allocation behaviors before and during the epidemic, and according to further multiple comparison analysis (Table 9), pre-epidemic risk averse vs. risk neutral risk-averse, risk-averse and risk-preferring allocations to Current/Fixed Deposit, Commercial insurance, Stocks/Funds, Options/Futures, Bonds, Overseas Assets, Precious Metals are statistically significantly but no significant difference between risk preferences and risk neutrals. The results in the middle and late stages of the epidemic are the same as the pre-epidemic results, i.e., there are statistically significant differences in household financial asset allocations between risk averse and risk neutral and risk averse and risk preferences, but not between risk preferences and risk neutrals. In addition, we performed median descriptive statistics on the household financial asset allocation profiles of different risk attitudes, and the results are shown in Table 10, where the share allocated to Current/Fixed Deposit for risk preferences is significantly smaller than the median for risk averse (26.5, 23, 22) in the middle and end of the pre-epidemic period (59.5, 60, 60), while we found that risk Neutrals and risk-averse have essentially equal medians in the pre-epidemic period (26, 26.5).

**Table 8:** Analysis of the differences in household financial asset allocation by different risk attitudes.

**Time**	**Items**	***K-W*-test** **statistic *H*-value**	***P*-value**
Before the COVID-19	Current/fixed deposit	48.29	<0.001
	Commercial insurance	30.17	<0.001
	Stocks/funds	48.35	<0.001
	Options/futures	42.92	<0.001
	Bonds	33.37	<0.001
	Overseas assets	23.90	<0.001
	Precious metals	15.68	<0.001
During the COVID-19	Current/fixed deposit	43.52	<0.001
	Commercial insurance	35.86	<0.001
	Stocks/funds	60.60	<0.001
	Options/futures	43.97	<0.001
	Bonds	30.79	<0.001
	Overseas assets	20.83	<0.001
	Precious metals	8.00	0.018
After the COVID-19	Current/fixed deposit	37.52	<0.001
	Commercial insurance	40.12	<0.001
	Stocks/funds	53.49	<0.001
	Options/futures	29.08	<0.001
	Bonds	30.03	<0.001
	Overseas assets	28.42	<0.001
	Precious metals	12.83	0.002

**Table 9:** A test of the variability of different risk attitudes on household financial asset allocation before the epidemic.

**Items**	**Risk attitude**	**Bonferroni** **Adjusted *P*-value**
Current/fixed deposit	2–1	1.000
	2–3	0.000
	1–3	0.000
Commercial insurance	3–1	0.000
	3–2	0.000
	1–2	1.000
Stocks/funds	3–1	0.000
	3–2	0.000
	1–2	0.993
Options/futures	3–1	0.000
	3–2	0.000
	1–2	1.000
Bonds	3–1	0.011
	3–2	0.000
	1–2	0.180
Overseas assets	3–1	0.002
	3–2	0.000
	1–2	1.000
Precious metals	3–2	0.002
	3–1	0.001
	2–1	0.892

1 for risk preferences, 2 for risk neutrals, 3 for risk averse.

**Table 10:** Description of median household financial asset allocation with different risk attitudes.

**Time**	**Items**	**Median value (P25,P75)**		
		**Risk preference**	**Risk-neutral**	**Risk-averse**
Before the COVID-19	Current/fixed deposit	26.5 (14.0, 51.5)	26.0 (15.8, 47.0)	59.5 (22.0, 100.0)
	Commercial insurance	12.0 (3.0, 20.0)	12.0 (3.0, 21.0)	4.0 (0.0, 18.0)
	Stocks/Funds	12.0 (4.0, 20.0)	13.0 (5.0, 20.0)	0.0 (0.0, 15.0)
	Options/futures	5.0 (0.0, 13.8)	6.0 (0.0, 14.0)	0.0 (0.0, 5.0)
	Bonds	3.5 (0.0, 12.0)	7.0 (0.0, 13.0)	0.0 (0.0, 8.3)
	Overseas assets	0.0 (0.0, 10.0)	0.0 (0.0, 10.0)	0.0 (0.0, 3.0)
	Precious metals	10.0 (0.0, 25.0)	7.0 (0.0, 20.0)	1.5 (0.0, 14.0)
During the COVID-19	Current/fixed deposit	23.0 (12.3, 60.0)	28.0 (15.0, 50.3)	60.0 (20.8, 100.0)
	Commercial insurance	10.0 (0.3, 20.0)	13.0 (4.0, 20.0)	0.0 (0.0, 16.0)
	Stocks/funds	11.0 (0.0, 24.0)	10.0 (3.0, 20.0)	0.0 (0.0, 10.3)
	Options/futures	2.0 (0.0, 14.0)	5.0 (0.0, 14.0)	0.0 (0.0, 3.3)
	Bonds	6.0 (0.0, 14.0)	6.0 (0.0, 14.0)	0.0(0.0,7.0)
	Overseas assets	0.5 (0.0, 11.0)	0.0(0.0, 11.0)	0.0 (0.0, 4.0)
	Precious metals	8.0 (0.0, 20.0)	7.0(0.0, 20.0)	2.5 (0.0, 18.3)
After the COVID-19	Current/fixed deposit	22.0 (12.0, 59.5)	26.0 (14.0, 50.0)	60.0 (18.0, 100.0)
	Commercial insurance	11.0 (3.0, 20.0)	12.0 (3.0, 20.0)	0.0 (0.0, 15.3)
	Stocks/funds	10.0 (0.0, 18.0)	12.0 (2.0, 20.0)	0.0(0.0,12.3)
	Options/futures	5.0 (0.0, 13.0)	5.0 (0.0, 13.0)	0.0 (0.0, 7.0)
	Bonds	4.0 (0.0, 12.0)	5.0(0.0, 14.0)	0.0 (0.0, 8.0)
	Overseas assets	0.0 (0.0, 12.8)	0.0 (0.0, 10.0)	0.0 (0.0, 2.0)
	Precious metals	10.0 (0.0, 21.0)	9.0 (0.0, 20.0)	0.0 (0.0, 16.0)

## Discussion

The Chinese government has implemented a “dynamic zero” policy, which imposes restrictions on residential travel activities and corporate production. In this study, we examine the financial asset allocation of Chinese households before, during, and after the COVID-19 epidemic. The results show that households' financial assets are more biased toward demand/fixed deposits and have a significantly higher incentive to save preventively during the pandemic compared to before the COVID-19 epidemic (Leland, 1968; Deaton, 1989), but then decrease significantly after the pandemic, investing their assets in stocks/funds and precious metals to increase the earning effect. In addition, our data show that different gender, education, occupation, and annual income have an impact on risk attitudes and whether uncertainty makes a change.

The current findings are consistent with the theory of certainty effects (Kahneman and Tversky, 2012), and we find that the most significant difference is in the wealth allocation to equity/fund-based products before and after the epidemic, suggesting that residents, faced with the prospect of income uncertainty, do not choose to risk allocating their assets to risky products, but instead allocate them to cash and its equivalents (demand/ fixed deposit type), increasing the precautionary motive. However, after the end of the epidemic and experiencing the impact of falling incomes, household wealth is allocated partly from savings to high-risk and high-return products, such as the stock/fund category, options/futures category, and precious metals products.

Unlike the expected increase in commercial insurance in the middle and late stages of the pandemic, there was no difference in the allocation of commercial insurance to household financial assets before and after the epidemic, a phenomenon that may be related to the distrustful attitude of Chinese residents toward insurance (Tang et al., 2013). Fixed-income products such as bonds also did not increase significantly during the pandemic, but instead declined, related to the large fluctuations in debt market yields during the pandemic (Deng, 2021) and to the increased risk of return uncertainty due to the weak liquidity of bonds (Gubareva, 2021), with the share of bond products remaining essentially the same as pre-pandemic allocations after the end of the pandemic.

Also in this survey, we note that differences in attitudes toward risk across socio-demographic characteristics lead to differences in whether uncertainty about economic and social developments will change financial asset allocation: women are more likely to change their original decisions in uncertain situations, while men are less likely to do so, with more risk-averse men and more risk-averse women. The higher the level of education, the more risk-averse and more likely to change their decisions in response to social changes. The more stable the occupation the more risk-neutral and risk-averse people are, and also choose to change their decisions in the face of uncertainty scenarios. Similarly the higher the income, the more they will change their choices, but they are more risk-neutral and risk-averse.

Different risk attitudes lead to different household asset financial allocation behaviors and influence residents' preferences for household financial asset allocation portfolios. Risk-averse individuals exhibit significantly more aggressive allocation behaviors than risk-averse individuals in the pre-pandemic and mid-pandemic periods, and the allocation share of savings products is significantly smaller than that of risk-averse individuals. In addition, we find that the household asset allocation portfolios of risk averse individuals are highly homogenized, with Current/Fixed Deposit, Commercial insurance, and Precious Metals in the pre-pandemic asset allocation portfolio and only Current/Fixed Deposit and Precious Metals in the mid-pandemic asset allocation portfolio. We explain this finding by suggesting that most households have a weak risk tolerance and that the main function of household wealth is to meet household precautionary and daily expenditure needs. In times of income uncertainty, safe and liquid assets are used as “safety buffers” by risk averse households, and risk averse households are more risk averse and require larger safety buffers and are therefore less likely to own risky investments (Barasinska et al., 2012).

There are three limitations of this survey study that should be considered. One is the issue of sample size and capacity; we only surveyed the Chinese population, which is influenced by differences in race, institutions, and perceptions (Krosnick and Milburn, 1990; Seemann et al., 2004; Ng et al., 2021), which may lead to findings that are applicable only in China, so we encourage international research on the impact of COVID-19 on household financial asset allocation; second, the question of the impact on household financial asset allocation during the epidemic, there is a need for follow-up surveys to determine whether household financial asset allocation will change when the epidemic rebounds again compared to the first pandemic. Third, the reliability of the questionnaire assessment, as it involves the household asset allocation situation, there may be cases of false answers, and if this happens it will seriously affect our findings, but the results of this survey are more consistent with the results of existing studies (Leland, 1968; Deaton, 1989).

## Conclusion

This study uses Chinese survey data to fill the gap that the pandemic affects household financial asset allocation through psychological factors, and explores the differences in household financial asset allocation behavior of people with different risk attitudes before, during and after the pandemic, which helps to enrich the literature on psychological expectations and behavioral choices under uncertainty scenarios.

The current findings suggest that, the COVID-19 pandemic period, when enterprises shut down their production, caused an increase in uncertainty about residents' income and a change in their psychological expectations about future economic and social development, leading to an increase in precautionary savings during the pandemic, and a part of the savings was invested in high-risk and high-return products to increase income after the pandemic ended, and household financial asset allocation before and the allocation of household financial assets changed continuously during the mid- and post-pandemic period. The choice of household financial asset allocation in the post-pandemic era varies among people with different risk attitudes, with risk averse people being more conservative in their asset allocation behavior in the post-pandemic period. In the future, households should develop asset allocation plans and allocate assets rationally to prevent the occurrence of unexpected events that lead to large fluctuations in household assets.

## Data availability statement

The original contributions presented in the study are included in the article/supplementary material, further inquiries can be directed to the corresponding author/s.

## Ethics statement

Ethical review and approval was not required for the study on human participants in accordance with the local legislation and institutional requirements. Written informed consent from the participants' legal guardian/next of kin was not required to participate in this study in accordance with the national legislation and the institutional requirements.

## Author contributions

HJ, SF, and MX were involved in the questionnaire design, survey, literature review, data compilation and analysis, and article writing for this study. All authors contributed to the article and approved the submitted version.

## Conflict of interest

The authors declare that the research was conducted in the absence of any commercial or financial relationships that could be construed as a potential conflict of interest.

## Publisher's note

All claims expressed in this article are solely those of the authors and do not necessarily represent those of their affiliated organizations, or those of the publisher, the editors and the reviewers. Any product that may be evaluated in this article, or claim that may be made by its manufacturer, is not guaranteed or endorsed by the publisher.
